# Late cerebrospinal fluid drainage for paraplegia developed following Bentall procedure for acute type a aortic dissection

**DOI:** 10.1186/1749-8090-8-S1-O26

**Published:** 2013-09-11

**Authors:** A Kucuker, M Hidiroglu, T Gumus, L Cetin, H Bayram, E Sener

**Affiliations:** 1 Ataturk Training and Education Hospital, Cardiovascular Surgery Department, Izmir, Turkey; 2 Ataturk Training and Education Hospital, Anesthesiology and Reanimation Department, Izmir, Turkey

## Background

Paraplegia is not a common complication following surgery for acute type A aortic dissection. Therefore cerebrospinal fluid drainage is not a routine practice perioperatively. We are presenting a case that developed late paraplegia, which resolved with cerebrospinal fluid(CSF) drainage 8 days after the operation.

## Methods

A 51 year old woman who had chest and back pain and syncope, was admitted to emergency department. Thorax CT revealed acute type A aortic dissection and she underwent emergency surgery. Modified Bentall procedure along with hemiarch replacement was performed using antegrade selective cerebral perfusion. The next day she developed cardiac tamponade and ischemia of the left leg. Re-exploration did not reveal any active bleeding source and right-to-left femoro-femoral crossover bypass was performed with a 10 mm dacron graft during repeat sternotomy. Distal pulses were palpable for both legs after the procedure. On third postoperative day she required a second re-exploration for bleeding and tamponade. Due to hemodynamic instability she received inotropes, required prolonged sedation and dialysis. On postoperative day 5, sedation was terminated and she was extubated but her physical examination revealed paraplegia. Medical theraphy was started.CSF drainage was performed afterwards.

## Results

Following 24 hours of CSF drainage she began moving her legs. On the follow-up period, she was able to stand-up with support. Renal functions also recovered and she was out of ICU on postoperative day 10.She was discharged on day 17 to a physical therapy unit. She is now on postoperative18 months with no walking disability.

## Conclusion

Delayed paraplegia can be observed after descending aorta interventions and there are many reports of recovery following reinstitution of CSF drainage. Late paraplegia is however rare after type A aortic dissection surgery. Our case demonstrates that late CSF drainage can also be dramatically therapeutic in such a situation.

